# Cyberspace and Libel: A Dangerous Balance for Physicians

**DOI:** 10.2196/22271

**Published:** 2021-05-27

**Authors:** Varsha Chiruvella, Achuta Kumar Guddati

**Affiliations:** 1 Medical College of Georgia Augusta University Augusta, GA United States

**Keywords:** libel, reputation, physician, law, legal, defamation

## Abstract

Freedom of speech and expression is one of the core tenets of modern societies. It was deemed to be so fundamentally essential to early American life that it was inscribed as the First Amendment of the United States Constitution. Over the past century, the rise of modern life also marked the rise of the digital era and age of social media. Freedom of speech thus transitioned from print to electronic media. Access to such content is almost instantaneous and available to a vast audience. From social media to online rating websites, online defamation may cause irreparable damage to a physician’s reputation and practice. It is especially relevant in these times of political turbulence where the battle to separate facts from misinformation has started a debate about the responsibility of social media. The historical context of libel and its applicability in the age of increasing online presence is important for physicians since they are also bound by duty to protect the privacy of their patients. The use of public rating sites and social media will continue to be important for physicians, as online presence and incidents of defamation impact the practice of medicine.

## Introduction

After many wars fought and won for freedom, Americans became free people living in a country under a democratically elected government. Although the government has control over civil conduct, its legitimate state power is seemingly unable to touch one monumental aspect of American lives—freedom of speech and expression. By definition, libel is a form of defamation conveyed by written text, pictures, signs or other physical forms of communication. It is detrimental to a person’s reputation, personal or professional, and exposes them to public contempt or ridicule [1]. However, protection offered from the First Amendment has given the public a legal platform that facilitates discourse on current topics, on which ideas and opinions can be exchanged, and that offers protection against defamation in certain circumstances. The First Amendment states

Congress shall make no law respecting an establishment of religion, or prohibiting the free exercise thereof; or abridging the freedom of speech, or of the press; or the right of the people peaceably to assemble, and to petition the Government for a redress of grievances.

2

While the text explicitly states limitations applicable to Congress, the First Amendment has also been interpreted to encompass all branches of government, including federal, state, and local. This is the textual basis for the state action requirement that a plaintiff must demonstrate that local, state, or federal government sectors were responsible for a violation [3]. The United States Supreme Court ruling from the 1964 court case New York Times Co. v. Sullivan [4] added that it was prohibited that a public official receive financial recovery from a defamatory falsehood, unless it was proven that the statements were made with malice or reckless disregard [4,5]. This was a landmark case pertaining to freedom of press protection by the First Amendment which was later adopted in nonmedia defendant cases. It is also important to distinguish defamation from satire. Satire is a literary form of criticism that mocks and ridicules, commonly seen in political commentary shows and used by political cartoonists or comedians to criticize public figures. Satire is implicitly protected by the First Amendment, under the free expression clause. Unlike libel, satire is not to be understood in a literal sense. However, like libel, satire can frequently be the topic of legal discussion.

## Libel: A Historical Perspective

Libel has been a part of written communication for centuries. History provides plenty of antiquated, yet relevant, case examples of libel that sparked future discussions on the ethics of professional medical libel. During the 1793 yellow fever epidemic in Philadelphia, William Cobbett, a British journalist, published his concern over an American doctor’s techniques to treat yellow fever in many papers. Dr. Benjamin Rush combatted the epidemic through mercury-based purgative and aggressive bloodletting—an approach largely discredited as a means of treatment later on in the 19th century. Although Cobbett was not a medical professional, he was a frontrunner in the application of medical epidemiology and biostatistics. Heralding evidence-based medicine, Cobbett used municipal records to prove that the perceived ghastly interventions performed by Rush did not in fact decrease the death rate from yellow fever. He presented data on mortalities during the epidemic, reporting that in the month following Rush’s implemented treatment regimens, there was an average of 67 deaths per day [6]. While the use of data was revolutionary and his numbers did speak for themselves, Cobbett was a journalist and used his most powerful arsenal against Rush: the written word. Repeated published attacks against the doctor and his medical practice were publicly viewed and responded to, eventually leading to a medical libel lawsuit filed by Rush. In one text, Cobbett wrote,

...a mosquito, a horse-leach, a weasel – all are bleeders and understand their business full as well as Rush does his.

7

Rush openly said that such inflammatory texts had compromised his business and diminished his patient’s confidence in his medical profession. After years of back-and-forth slander and trial hearings, Rush succeeded in his suit.

Through a contemporary lens, this result would be improbable given the added requisites in the United State Constitution for a case of libel. In the famous 1964 case New York Times Co. v. Sullivan [4], proof of actual malice was made a requirement to award of damages in a libel suit involving public figures. Justice William Brennan famously wrote that America has a

...profound national commitment to the principle that debate on public issues should be uninhibited, robust, and wide-open, [although] it may well include vehement, caustic, and sometimes unpleasantly sharp attacks on government and public officials.

4

The Court reasoned that open debate on public official conduct was more important than the potential damage to officials’ reputations. A *public figure* is legally defined an individual of great public interest or fame such as politicians, celebrities, and well-known athletes. The term is commonly used in libel and defamation cases in which the standard for evidence is relatively higher, as proof that the remarks were published with *actual malice* is necessary. Proof of actual malice means that the publisher either knew that the statement was false or acted with reckless disregard for whether it was true or not. For example, one individual well-known for his revolutionary dieting advice, Dr. Robert Atkins, was considered a public figure in the 1975 court case Atkins v. Friedman [8], for he had sold millions of copies of his book Dr. Atkins Revolutionary Diet. A public figure under the legal tenet of actual malice, Atkins could not be compensated

...in the absence of proof that the defendant published the item with knowledge of its falsity or in reckless disregard of the truth.

8

Several additions and modifications to the court standing on New York Times Co. v. Sullivan have taken place in the years following the initial ruling. The 1974 court case Gertz v. Robert Welch, Inc. [9] was an exception to the precedent set by New York Times Co. v. Sullivan, stating that actual malice was not necessary for a case of defamation of private person if negligence is present. The Court rationalization is that public, unlike private, figures assume the risk of being attacked due to voluntarily entering the public light and must be prepared to face some attack. Furthermore, public figures have means of self-help and media to combat reputational harm that private persons simply cannot take advantage of to the same extent [10]. The second category of public figure is called the *limited purpose* public figure. Cited in the case Gertz v. Robert Welch, Inc., these individuals are those who have

...thrust themselves to the forefront of particular public controversies in order to influence the resolution of the issues involved.

9

Limited purpose public figures not only include individuals who shape debate on particular public issues and utilize media for influence, but also those who have distinguished themselves in a particular field. Just as famous basketball players in the National Basketball Association are considered public figures of the field of basketball, physicians can be considered limited purpose public figures, as they are especially distinguished in the field of medicine. However, the actual malice standard applies to both public figures and limited purpose public figures if the subject matter or controversy in question is related to the field in which the individual is prominent [11].

Unlike the ease with which Rush had filed a medical libel lawsuit in the 1700s, a professional in the modern day has to meet higher legal standards to establish a defamation action. Now, Cobbett’s published words would simply be labeled as an opinion rather than as libel to which a case of defamation is applicable. As outlined in New York Times Co. v. Sullivan ruling, a plaintiff must show falsity in the statement of fact made, that it was defamatory and published, that an injury resulted from the publication, and that the defendant acted with a degree of fault. While private persons need not show proof of actual malice, negligence must be demonstrated. Like public figures, limited purpose public figures such as physicians must demonstrate actual malice. In addition, many websites that enable internet defamation, such as physician rating sites, are insulated against litigious claims from doctors under Section 230 of the 1996 Communication Decency Act [12], which makes it more challenging to sue a web-based platform for defamation. This law states:

No provider or user of an interactive computer service shall be treated as the publisher or speaker of any information provided by another information content provider

12

If the physician can identify the author, they could file a case against the author; however, the cloak of anonymity often falls over commenters and reviewers on the internet, adding additional obstacles for physicians. These tenets provide the public means to express free speech and protect the principle that debate on public issues should be uninhibited, despite the potential caustic repercussions on individuals. Therefore, given these exacting requirements, physicians may find it difficult and may be unsuccessful in pursuing litigation for libel.

## Libel in the Age of Social Media

While the 18th century had its share of defamation through newspaper writings, the 21st century introduced libel in the vast world of social media. With the invention of the internet and social media, people around the world can write what they experience, witness, and believe in a completely public arena. But that raises the question whether a tweet or public post on social media directed toward a medical professional constitutes libel. Is it considered libel if one were to express criticism about certain physician practices? The reality is that most people use the internet as a means to obtain information on health care professionals. One study [13] from 2005 shows that 80% of patients reported using the internet to research health topics such as specific medical conditions and prescription drug. Furthermore, a 2007 survey found that approximately one-third of internet users in California employ it for the purposes of finding a physician in a health network as well as for finding ratings of physicians on websites [14]. Another study [15] similarly notes that 24% of 61% of adults in the US who look online for health information have also looked online for ratings or reviews of doctors or other providers. More recent studies [16,17] have found that medically related internet searches were most related to symptom exploration. Subsequent reading about certain medical, and possibly unrelated, conditions without input from trained medical professionals may be a cause of acquiring and spreading false medical information. Recent reports have discovered that the internet can improve physician-patient relationships and communication, depending on the quality of information discussed [18]. Utilization of the internet for such health-related research could affect patient decision making when it comes to choosing their providers. Popular physician rating sites such as Vitals, Yelp, Angie’s List, Healthgrades, RateMDs, and Zagat have become increasingly integrated into our lives and essential to assess before choosing care under a medical professional. With approximately 90% of physicians’ professional information accessible online, it is no surprise that individuals use these platforms to write reviews, albeit most are positive [19]. A 2010 study [20] on online evaluations of physicians reported that, based on 33 physician-rating websites, 88% of reviews were positive in nature, while only 6% were negative.

Given these results, web-based platforms for physician ratings tend to be more beneficial than harmful, providing resources for patients and mostly positive feedback for providers. Physician-rating platforms are windows for individuals to report their experiences of a medical professional. If one considers how most individuals use rating platforms and the seemingly positive nature of most reviews, the chance of discovering anonymous criticism that is considered libel is slim. Furthermore, it would be legally difficult for a physician to file a case of libel, given the many requisites for this claim. First Amendment protections are broad, so rigorous requirements needed to be imposed to prove libel and genuine defamation of individuals, including state action requirements. As previously stated, per New York Times Co. v. Sullivan [4], in order for comments to satisfy the legal definition of defamation, they would need to be false (ie, lacking justification), communicated to a third party, and damaging to the injured party’s reputation [21].

However, this may not always be the circumstance for cases of defamation. Defamation *per se* need not require evidence of harm to an individual for proving online defamation. In contrast to defamation *per quod*, where false statements are not inherently defamatory, defamation *per se* applies to false statements that are considered so damaging that they are deemed defamatory. While damage and actual malice must be proven in defamation *per quod*, statements are presumed harmful for defamation *per se* if false allegations fall into one of 4 categories: indication of involvement in criminal activity; indication of contagious, transmittable, and infectious disease; indications of heinous acts or sexual misconduct; and indications of behavior incompatible with managing professions, business, or trade [1]. Nonetheless, proving defamation is not easy, as statements that are considered opinions are not defamatory in the eyes of the law. In fact, negative comments on physician-rating websites qualitatively address physician interpersonal relationships, bedside mannerisms, and staff behavior [22]. Rather than illuminating aspects of the professional’s medical expertise, the majority of online reviews place heavy emphasis on nonclinical attributes, such as office waiting time, etc. On the contrary, defamatory statements include false comments like that a physician is not board certified or other allegations that fall into the 4 previously mentioned categories. Furthermore, false allegations made online may be anonymously posted. In this case, physicians can file “John Doe” lawsuits. After demonstrating a prima facie case for defamation, a subpoena can be filed to track the internet protocol address to determine the identity of the poster.

While it is difficult for physicians to start a defamation lawsuit, it is not impossible. This does, however, come at a cost both for the physician on trial and for the defendant, the creator of the libelous statement. An abundance of time and expensive legal fees are just a few hurdles that both parties face. If the individuals responsible for the defamation are found guilty, they may face tremendous fees and could lose their employment [1]. One such case is that of Dr. Pieter Cohen versus Hi-Tech Pharmaceuticals from 2017 [23]. Dr. Cohen, an assistant professor at Harvard Medical School, had published a peer-reviewed scientific article revealing the toxic components of weight-loss supplements manufactured by Hi-Tech. The company consequently filed suit for libel against Cohen. After 6 months of trials, the court ultimately ruled in favor of Cohen. Although he had won the lawsuit, it had left him with over $7000 in legal fees and the lost, irreplaceable time spent battling the pharmaceutical company rather than conducting research [23]. Dr. Cohen was one of the more fortunate individuals fighting a defamation lawsuit, as he had the financial support and occupational backing of Harvard Medical School. Others who make controversial claims as part of nonprofit organizations and small private institutions may, however, bear the brunt of such burdens. Observing this case from the lens of patients can explain why many may hesitate to express their thoughts online out of fear of repercussion from their medical providers, who, like Dr. Cohen, may be associated with large hospitals.

This, however, does not stop all patients from commenting on their provider’s performance. It certainly would not be surprising if, of all medical practitioners, plastic surgeons received the most piercing online reviews in an age of aesthetic modification. In fact, one study [24] reports that, from 2011 to 2016, the number of online reviews on Google, Yelp, and RealSelf for breast augmentation grew at an average of 42.6% per year, with 69.5% of reviews commenting on aesthetic outcomes. One prominent case from 2014 was Loftus v. Nazari. Dr. Jean Loftus was a plastic surgeon who had performed a breast augmentation, breast lift, arm lift, and tummy tuck on patient Catherin Nazari. Unhappy with the results of her operation, Nazari took her frustration online and posted several negative reviews of Dr. Loftus on 3 rating sites. She most notably wrote that she was “left with permanent nerve damage in both arms, severe abdominal pain, horrible scars, and disfigurement in both breasts” because of Dr. Loftus [25]. In response, Loftus filed a defamation lawsuit against Nazari. Unfortunately for the physician in this case, the courts claimed that Nazari’s remarks on her physical condition and Dr. Loftus’ negligence were her opinions, as the comments were published on opinion websites [25]. The case of Loftus v. Nazari is one of several examples of defamation lawsuits in which pejorative comments are viewed as protected opinions by law.

## The Libelous Arena of Twitter

The topic of libel in web-based platforms is also gaining more media attention following several recent tweets by government officials in the US. Most notably, Twitter labeled some of US President Donald Trump’s tweets as misleading and a violation of the company's rules about glorifying violence. Other tweets by Trump describing mail-in-voting as fraudulent resulted in the company’s placement of a fact-checking label on 2 of Trump’s tweets [26]. Although one may think that it is important to distinguish the specific platforms in which allegations are expressed, the Florida Bar Journal states that the nature of the medium, whether public or private, is not as important as the content and nature of the communication [27]. On the contrary, comparing libelous statements on both private company sites such as Twitter and public forums such as RateMDs is like comparing apples to oranges due to Twitter’s inability and RateMDs’ ability to moderate misinformation and libel and remove users as it sees fit. The following is the physician review site’s policy on ratings:

Reviews flagged for removal are reviewed by RateMDs and taken down if deemed inappropriate (for instance, because they contain demonstrably inaccurate or out of date information (to the extent that information was out of date at the time of the review), are libelous, or include accusations of unlawful activity, profanities or vulgarity, privacy violations, spam, or details that are not relevant or related to a patient’s visit).

28

This is in stark contrast to Twitter’s policy on the matter, which fosters discourse as long as it does not involve or incite violence, sexual exploitation, abuse, sensitive media, illegal services, and so on:

Twitter is a social broadcast network that enables people and organizations to publicly share brief messages instantly around the world. This brings a variety of people with different voices, ideas, and perspectives. People are allowed to post content, including potentially inflammatory content, as long as they’re not violating the Twitter Rules. It’s important to know that Twitter does not screen content or remove potentially offensive content.

29

While both RateMDs and Twitter foster online discourse, Twitter’s requirements and standard for removal of content are much more extreme in nature, as libelous claims made against physicians are not considered “offensive content” enough for the private company to delete.

But how is Twitter able to house potentially damaging or libelous allegations, whether written about a physician or by the president? Section 230 of the Provision of the Communication Decency Act of 1996 is responsible [30]. It does this by protecting big social media websites from being liable for user content. The section states that

...no provider or user of an interactive computer service shall be treated as the publisher or speaker of any information provided by another information content provider.

31

Essentially, the function of the Communication Decency Act is to shield internet service providers from third-party content on their platform. Altering or revoking the protection afforded by this law may have interesting consequences: Would web-based platforms ensure that posted content is factually correct or would they stipulate authors to declare that their submissions are opinions? Both scenarios have implications for the medical community since vetted content is likely to lack misinformation and enforcement of an opinion label on posts would likely decrease credibility.

## Solutions for Physicians

Patients are increasingly using web-based platforms to convey their views on medical providers. The challenge for physicians to build an adequate case for libel, and win, raises another question: How can medical providers develop strategies to counter claims? What is expected of them when they are faced with libel or simply negative comments online? While most commentary on physician review websites is not legally defined as libel, there are instances of online defamation. Some cases may even surge onto the news and widespread public platforms. Arguably, the most important aspect that a physician must keep in mind when defending their reputation is to maintain professionalism and patient-physician confidentiality. Due to patient privacy provisions in the 1966 Health Insurance Portability and Accountability Act (HIPAA) and patient-confidentiality laws, physicians generally cannot easily or legally repudiate caustic or false comments on public forums without violating a privacy regulation [21]. The HIPAA Privacy Rule protects all

...individually identifiable health information held or transmitted by a covered entity or its business associate, in any form or media, whether electronic, paper, or oral...

32

and is termed *protected health information* [33]. When faced with negative commentary online, it would be in the provider’s best interest not to respond, as posting reveals physician-patient relationship and violates HIPAA [34]. Nevertheless, HIPAA violations are rather common. The US Department of Health and Human Services reports that between April 2003 and September 2017, a total of 165,175 privacy rule complaints were received [34]. If a physician discusses or transmits protected health information without patient consent, they could face a financial penalty of up to US $50,000, depending on the nature and extent of the breach in confidentiality [35]. It is thus imperative for medical professionals, who choose to respond online to public scrutiny, to remain in accordance with HIPAA policies.

The question remains, what is a practical solution for physicians when faced with libelous claims? In addition to maintaining a professional physician-patient relationship and following HIPAA protocol, there are steps that medical professionals may take in the event of encountering defamation. Being proactive rather than reactive by monitoring and contacting public online spaces to remove the defamatory comments would be an appropriate step to take. Medical professionals should regularly check web-based platforms and set alerts to notify them of comments on their practice. Due to the tremendous financial cost and economic burden a medical practice may face with a defamation case, resorting to litigation should be approached with caution [1]. Physicians have the additional option of paying for reputation management software. For instance, RateMDs’ Promoted Plus and Promoted packages for $359/month and $179/month, respectively, allow physicians to hide up to 3 unfavorable comments on the site.

Some doctors even go to the extent of nondisclosure agreements (NDAs), asking patients to sign a legal document that waives their right to post unauthorized online reviews in order to prevent risk of physician defamation. Dr. Jeffrey Segal is the chief executive officer and founder of Medical Justice, an organization that supports the use of such waivers [36]. NDAs, commonly called confidentiality agreements, are binding contracts that govern the sharing of information between people and organizations and that set limits for information use. NDAs are widely used in the workspace as a means of creating confidential employer-employee relationships and have become the topic of discussion in the rise of the #MeToo movement, as NDAs may prohibit victims of sexual harassment or assault from publicly discussing settlements or their trauma [37,38]. Dr. Segal’s document, which has been adopted by several thousand providers and patients each year, states that physicians will provide additional privacy protection measures to patients in exchange for their agreement to not post positive or negative comments without the doctor’s assent. However, patients still have plenty of avenues to speak about their experiences with family, friends, and other individuals, and review committees. While this movement is in no way an effort to forbid negative reviews and is not an antilibel intention, it is an attempt to provoke discussion on self-policing websites [39].

## Physician Rating Through a Different Lens

Despite the negative perception that physicians may have of rating sites, online reviews and rating patterns could potentially help doctors improve or better manage their professional reputations. Given that studies show most reviews are positive and express opinions on physician attributes and overall satisfaction with the in-office visit, it would be expected that this online transparency should benefit providers [40]. This is the fundamental basis for the University of Utah health care system’s venture in 2012 to survey all patients and post all their comments online [41]. Harvard Business Review cites University of Utah as the first hospital system in the United States to post all online physician reviews and comments. This strategy allows providers to privately receive their patient-experience data, which has reportedly resulted in conversations on how to improve the organization’s approach to health care [42]. A physician can also receive a report card on their improvements, which patients in the system can review online due to implementation of transparent measures.

## Conclusion

The internet has become intertwined in the daily lives of individuals in all professions and of all ages. The increasing use of web-based discussion, commenting, and spreading of information regarding physicians, however, should be a topic of interest. With the increasing utilization of web-based platforms to comment on a physician’s practice, awareness of libel in the medical profession may grow as well. While filing a defamation claim may be enticing for a physician in sight of an inflammatory comment online, physicians should be aware of the difficulty, costs, risks, and requirements by law in pursuing such cases. Physicians do have several options on how to handle libelous claims, while, first and foremost, taking into consideration patient-physician confidentiality as outlined by HIPAA. It is also important to consider the use of web-based platforms and social media as an opportunity for improvement to medicine, rather than as an attack on their practice. Listening to online commentary may be able to help physicians acknowledge previously unrecognized faults or deficiencies and better understand patient perspectives. While the internet may hold offensive commentary or false allegations, it may also become the building block of strong physician–patient relationships. In conclusion, whether the internet poses an opportunity for one to discover more about a disease, research a particular doctor, or speak at length about an unpleasant experience, it is undoubtedly shaping the landscape of medicine.

## Abbreviations

HIPAA: Health Insurance Portability and Accountability Act
NDA: nondisclosure agreement
